# What Is True Halving in the Payoff Matrix of Game Theory?

**DOI:** 10.1371/journal.pone.0159670

**Published:** 2016-08-03

**Authors:** Hiromu Ito, Yuki Katsumata, Eisuke Hasegawa, Jin Yoshimura

**Affiliations:** 1 Department of International Health, Institute of Tropical Medicine, Nagasaki University, Nagasaki, Japan; 2 Graduate School of Science and Technology, Shizuoka University, Hamamatsu, Shizuoka, Japan; 3 Department of Mathematical and Systems Engineering, Shizuoka University, Hamamatsu, Shizuoka, Japan; 4 Laboratory of Animal Ecology, Department of Ecology and Systematics, Graduate School of Agriculture, Hokkaido University, Sapporo, Hokkaido, Japan; 5 Department of Environmental and Forest Biology, State University of New York College of Environmental Science and Forestry, Syracuse, New York, United States of America; 6 Marine Biosystems Research Center, Chiba University, Uchiura, Kamogawa, Chiba, Japan; Tianjin University of Technology, CHINA

## Abstract

In game theory, there are two social interpretations of rewards (payoffs) for decision-making strategies: (1) the interpretation based on the utility criterion derived from expected utility theory and (2) the interpretation based on the quantitative criterion (amount of gain) derived from validity in the empirical context. A dynamic decision theory has recently been developed in which dynamic utility is a conditional (state) variable that is a function of the current wealth of a decision maker. We applied dynamic utility to the equal division in dove-dove contests in the hawk-dove game. Our results indicate that under the utility criterion, the half-share of utility becomes proportional to a player’s current wealth. Our results are consistent with studies of the sense of fairness in animals, which indicate that the quantitative criterion has greater validity than the utility criterion. We also find that traditional analyses of repeated games must be reevaluated.

## Introduction

Recently, game theory has been studied extensively to investigate the reasons why cooperation evolved in human and animal society, e.g., networks and other spatial structures, heterogeneity in behavior or rationality, and punishments [1–13]. These developments introduce some complexities in game theory itself such that the game becomes more realistic. Instead, in this work, we focus on the nature of decision making in game theory. Note that decision theory and game theory are optimization theories of individual behaviors that have been systematized together. Decision theory is adopted when individuals face environmental uncertainty, whereas game theory applies when the behavior of other individuals is uncertain [14–15]. For these reasons, game theory is a special extension of decision theory.

The core of decision theory is referred to as expected utility theory, which maximizes the expectation value (average or mean) of utility [14, 16]. Based on the above relationship, the axioms of expected utility theory are used as the basic axioms of game theory. In this case, utility is a measure of content (satisfaction) if the given wealth (resource) is consumed. The utility of a given level of wealth is thus highly subjective and individualized; moreover, utility is therefore a dimensionless value without a unit, unlike monetary currencies (dollars and yen) and prison sentences (years). We introduce dynamic utility [17–18], a new concept of utility, in decision theory into game theory. Specifically, we consider half-sharing among doves in the hawk-dove game in terms of dynamic utility.

Game theory involves the study of individuals’ behavior in relation to other individuals in a population. The payoff matrix is the set of benefits for players against opponents. Therefore, the elements of the payoff matrix are understood as the values of utility. Thus, the social interpretations of strategies are understood to be based on the utility criterion [16, 19–20]. However, this utility interpretation frequently conflicts with the natural understanding of animal and human behaviors. Meanwhile, some people in economics actually use the amount of money (dollar value) in the payoff matrix because the amount of money (rewards) is not known in the utility criterion [21–30], but we like to know how many dollars we can obtain as a reward for victory. The main interest of game theory is in how people behave in a real-money game. To contrast real-money rewards with the utility criterion, we call the latter the quantitative criterion. Recently, several experimental studies have shown that animals behave according to the quantitative criterion when they are offered rewards after enduring suffering [31–34]. For example, if the same effort is required, animals complain strongly if the same amount of reward is not offered. Hence, animals are said to exhibit a sense of fairness, where fair division is defined as a half share of the resources, e.g., an ultimatum game [31–32, 34]. These observations seem to indicate that the quantitative criterion is more valid than the utility criterion.

Many techniques have thus adopted the quantitative criterion for payoff elements [21–29], leaving two alternatives for interpreting the elements of the payoff matrix in game theory: the utility criterion (dimensionless) and the quantitative criterion (with units, e.g., dollars).

Dynamic programming (DP) involves a numerical algorithm that solves for the optimal choice in sequential decision making. DP underlies the first true dynamic optimization model that was developed solely by Richard Bellman [35]. Later, stochastic control (theory) was developed but found to be mathematically equivalent to DP with additional complexity [21]. DP has been extensively applied to animal decision-making activity in behavioral ecology and has helped yield numerical solutions to various dynamic problems [36]. Despite these great achievements, theoretical understanding of the mechanisms of behavior is not provided by the numerical solutions yielded by DP.

Recently, a theory of dynamic utility optimization (DU) has been developed using the “Principle of Optimality,” the core principle of Bellman’s DP, which involves dynamic decision making under risk and uncertainty in which the growth rates of individual wealth are random variables that follow a simple stochastic process [17–18]. Because DU optimizes Markov chains (stochastic processes) as a form of sequential decision making, it maximizes the geometric mean of multiplicative growth rates.

Here, we explain the theoretical rationale of the dynamic utility model. The dynamic utility function is derived as follows [17–18]. Let time *t* = 0, …, *T* (final time) and *w*_*t*_, the wealth at *t*, be the non-negative state variables of a decision maker (independent, identically distributed random variables). Let *r*_*t*_ (>0) denote the multiplicative growth rate of the wealth at *t*, such that *w*_*t*+1_ = *r*_*t*_*w*_*t*_. Then, the wealth at *t*, *w*_*t*_, is expressed as

$$w_{t}=w_{0}r_{0}r_{1}r_{2}\cdots r_{t-1}$$ (1)

We assume that the growth rates *r*_*t*_ (*t* = 0, …, *T*) are independent identically distributed random variables that represent a stochastic process. The decision maker can optimize this stochastic process by choosing the best option at every time point in Eq (1). We thus maximize the final wealth at *T*, *w*_*T*_, such that

$$w_{T}\to \max$$ (2)

This maximization of the final wealth *w*_*T*_ (Eq (2)) is equivalent to maximization of the geometric mean growth rates such that

$$G(r)=\prod_{i=0}^{T-1}{r_{i}}^{\frac{1}{T}}:\to \max$$ (3)

Taking the logarithm of Eq (3), we obtain

$$\log\left\{G(r)\right\}=\frac{1}{T}\sum \log\left(r_{i}\right)=E\{\log\left(r\right)\}:\to \max$$ (4)

Eq (4) is rewritten in the form of utility theory in economics and operations research. We simply define utility function *u*(*r*) as

u(r)=logr (5)

and we maximize the expected utility E{*u*} [37]. Note that *w*_*t*+1_ = *r*_*t*_*w*_*t*_. Therefore, we obtain

$r_{t}=\frac{w_{t+1}}{w_{t}}=\frac{g_{t}+w_{t}}{w_{t}}$, where *g*_*t*_ is the gain at time *t*. Therefore, at any time *t*, we obtain

$$r=\frac{g+w}{w},$$ (6)

where *g* and *w* are the current gain and the current wealth, respectively. The growth utility formula (Eq (5)) is then further rewritten in the form of *g* (decision variable) given *w* (state variable) such that

$$u(g;w)=\log\left(\frac{g+w}{w}\right)$$ (7)

and we maximize the expected utility E{*u*(*g*;*w*)}, which indicates that the current wealth is the state variable for maximization of the final wealth. Therefore, this function *u*(*g*;*w*) (Eq (6)) violates the so-called independent axiom of the axiomatic system of utility theory [16]. Thus, the principle of optimality developed by Richard Bellman [35] contradicts with the traditional expected utility theory [16].

Thus, DU yields the following optimization principle:

$$Maximize:\;E\{u(g;w)\},\;where\;u(g;w)=\log\left(\frac{w+g}{w}\right)$$ (8)

Thus, the derived dynamic utility is in the form of a logarithmic function (Eq (8)). Note that the value of *g* satisfies–*w* < *g*. This analytical solution for DU demonstrates that the utility function depends on the current gain/loss (the decision variable) and the current wealth status (state variable) at the time of decision making. In the present study, we demonstrate that the traditional application of expected utility theory and game theory in behavioral studies is valid only as a static model.

By combining the maximization of future wealth and the avoidance of bankruptcy (the two optimization criteria), we obtain the following:

$$Maximize:E\{u(g;w)\},\;where\;u(g;w)=(1+f(g))\;\log\;\left(\frac{g+w}{w}\right)$$ (9)

where

$$f(g)=\left\{ \begin{array}{c} \;0\;\text{if}\;g\geq 0\\ cg\;\text{if}\;g<0 \end{array}\right.$$ (10)

Here, *c* is a constant. In the present study, the dynamic utility function (DUF) *u*(*g*;*w*) is applied to game theory; notably, the DUF avoids the arbitrariness of utility by mathematical derivation from DP. Using numerical analyses of some examples, we demonstrate that there are serious contradictions in the social interpretation of strategy when the utility criterion is used. These results indicate that traditional interpretation of game theory is a static optimization model that is valid only when all the players have equal current wealth. It cannot be applied to any game in which the current wealth of players varies over time; thus, the utility criterion cannot be applied to repeated games in which the current status of a player changes over time. In contrast, the quantitative criterion does not invoke a sense of unfairness in dividing the reward for games. Thus, we suggest that the quantitative criterion is more adaptable to the social interpretation of strategy in game theory than the utility criterion.

## Model and Results

**Fig 1:**
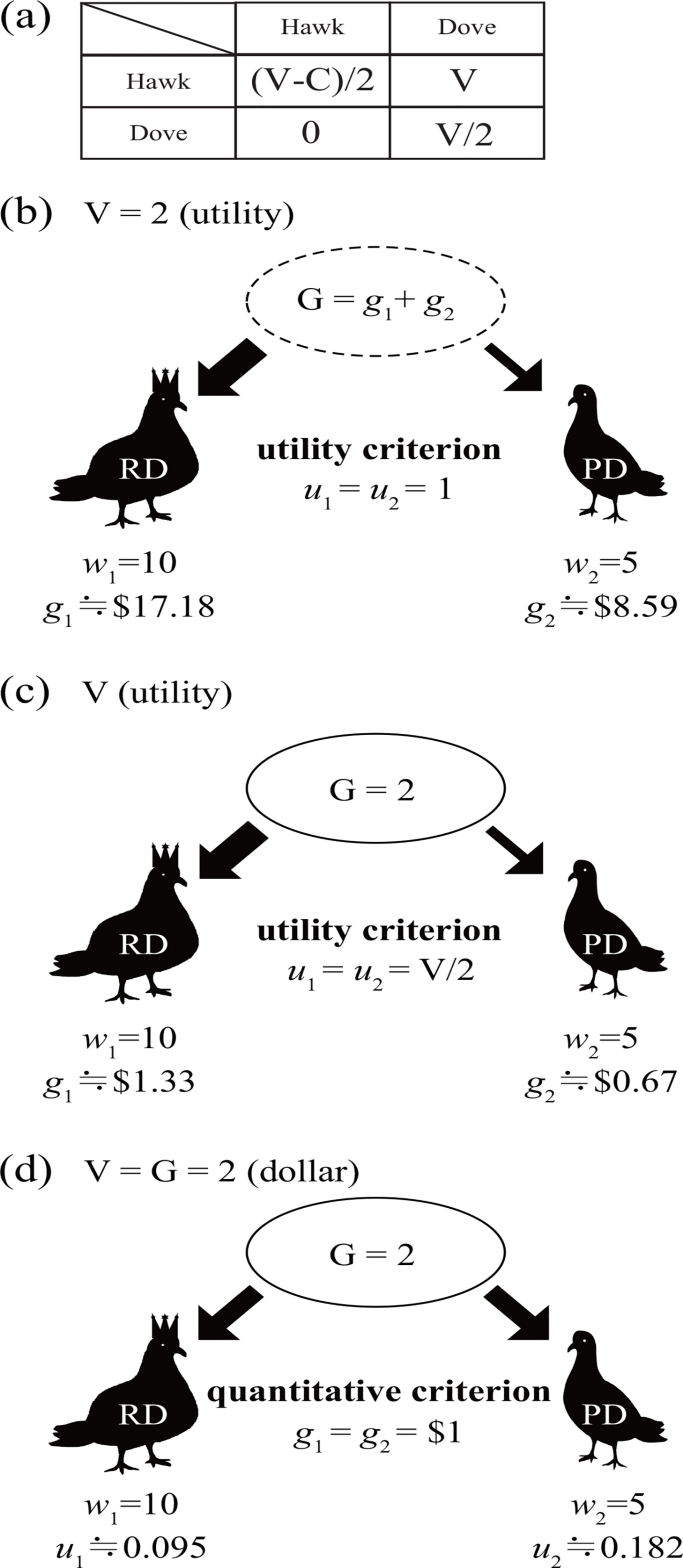
The halving of the victory reward based on the utility and quantitative criteria in the hawk-dove game. (a) Payoff matrix of the hawk-dove game, where V and C are the victory reward and fighting cost, respectively. (b, c, d) The halving outcomes of victory rewards (V) by two players adopting the dove strategy in which the current wealth of player 1 (rich dove; RD) and player 2 (poor dove; PD), *w*_1_ and *w*_2_, are *w*_1_ = 10 and *w*_2_ = 5. (b) The utility criterion in which V = 2 (utility) is divided by half, such that *u*_1_ = *u*_2_ = 1. The amount gained by each player is proportional to the player’s current wealth, such that $g_{i}=w_{i}(e^{u_{i}}-1)$. The total amount, G, of victory reward, V, varies based on the sum of the current wealth of both players, such that G = (*w*_1_ + *w*_2_)(*e*– 1). (c) The utility criterion in which the amount of reward G is set constant (G = $2). The gains of the players depend on the proportion of players’ current wealth, such that *g*_1_ = {*w*_1_/(*w*_1_ + *w*_2_)}G. The utilities of the two players are equal, but the amounts of gains differ based on the ratio of current wealth, as in (b). (d) The quantitative criterion in which V = G = 2 dollars. The utility of players depends on the current wealth of players, such that *u*_*i*_ = log {(*g*_*i*_ + *w*_*i*_)/*w*_*i*_}.

As an example of game theory, we consider the hawk-dove game (V: victory reward; and C: fighting cost), in which the sense of fairness appears when both players adopt the dove strategy (D, D) in their payoff matrix (Fig 1A) [14]. Here, the “dove” player against a dove opponent gains V/2 in every contest.

We apply the analytically derived DUF *u*(*g*;*w*) *=* log{(*g*+*w*)/*w*} to the case of fair division of victory reward (V/2) in (D, D). Numerically, we set *w*_1_ = 10 and *w*_2_ = 5 (unit: dollar). We compare the values of V/2 between the utility and quantitative criteria.

In the case of the utility criterion, both players acquire *u* = V/2 (unit: utility). The utility of each player *i* (*i* = 1, 2) should satisfy the following relationships between the current gain *g* and current wealth *w*:

$$u(g_{i};w_{i})=\log\left(\frac{g_{i}+w_{i}}{w_{i}}\right)\;\Leftrightarrow \;g_{i}(u=V/2;w_{i})=w_{i}(e^{V/2}-1)$$ (11)

Eq (11) results in the following serious flaws. The face-value of money that each player obtains depends on the relative wealth of the player, such that

$$u_{1}=u_{2}\;(=V/2)\;\Leftrightarrow \;\log\;\left(\frac{g_{1}+w_{1}}{w_{1}}\right)=\log\;\left(\frac{g_{2}+w_{2}}{w_{2}}\right)\;\Leftrightarrow \;g_{1}=\frac{w_{1}}{w_{2}}g_{2}$$ (12)

Therefore, halving the reward depends on the relative amount of the players’ current wealth *w*_1_/*w*_2_. For example, if we set V = 2 (utility), such that *u*_1_ = *u*_2_ = 1 (Fig 1B; case 1 in Table 1), then we obtain the following:

$$\left\{ \begin{array}{c} g_{1}(u_{1}=1;w_{1})=w_{1}(e^{u_{1}}-1)≅17.18\;[\$]\\ g_{2}(u_{2}=1;w_{2})=w_{2}(e^{u_{2}}-1)≅8.59\;[\$] \end{array}\right.$$ (13)

**Table 1:** Comparison of variables among three cases.

case	Criterion	Utility *u*	Gain *g*	Total gain G	Fig 1
1	utility	*u*_1_ = *u*_2_	*g*_1_ ≠ *g*_2_	variable	(b)
2	utility	*u*_1_ = *u*_2_	*g*_1_ ≠ *g*_2_	constant	(c)
3	quantitative	*u*_1_ ≠ *u*_2_	*g*_1_ = *g*_2_	constant	(d)

Thus, the richer the player, the greater the share that he or she should obtain in the equal-utility division (Fig 1B).

In case 1 (Table 1; Fig 1B), we also face the problem of the total amount G (= *g*_*1*_ + *g*_2_) of competitive resources. From Eq (11), the total resource G becomes proportional to the sum of the current wealth of both players. Therefore, G is large in games between rich players but small in those between poor players, which leads to the following logical inconsistency. In nature, and even in a society, competition occurs for existing resources, which indicates that the game rewards should be set equal to a constant prior to the beginning of a game. However, in case 1, the total reward G cannot be determined until who plays the game is determined. Furthermore, G may increase indefinitely in some repeated games when the sum of the current players’ wealth increases indefinitely. Thus, G should be a constant that is unaffected by players’ current wealth. We can avoid this problem of case 1 as follows. By setting a constant G in the utility criterion (case 2 in Table 1; Fig 1C), we can satisfy *u*_1_ = *u*_2_. Then, player 1 receives $g_{1}=\frac{w_{1}}{w_{1}+w_{2}}G$ as a reward. However, even in this case, the reward of a player becomes proportional to the current wealth of the players (as with case 1). Therefore, quantitative fairness is also not satisfied in this case.

In contrast, if we apply the quantitative criterion (Fig 1D; case 3), then each player obtains the same amount of money, such that *g* = V/2 (unit: dollar). In this case, the utility of each player *i* (*i* = 1, 2) depends on their current wealth, *w*_*i*_:

$$u_{i}(g=V/2;w_{i})=\log\;\left(\frac{V/2+w_{i}}{w_{i}}\right)$$ (14)

Thus, equal division of money results in a difference in the utility values of the players unless their current wealth is identical. For example, if we set V = 2 (unit: dollar), then *u*_1_ ≅ 0.095 and *u*_2_ ≅ 0.182 (Fig 1C).

$$\left\{ \begin{array}{l} u_{1}(g_{1}=1;w_{1})=\log\;\left(\frac{1+w_{1}}{w_{1}}\right)≅0.095\\ u_{2}(g_{2}=1;w_{2})=\log\;\left(\frac{1+w_{2}}{w_{2}}\right)≅0.182 \end{array}\right.$$ (15)

All three cases are summarized in Table 1.

**Fig 2:**
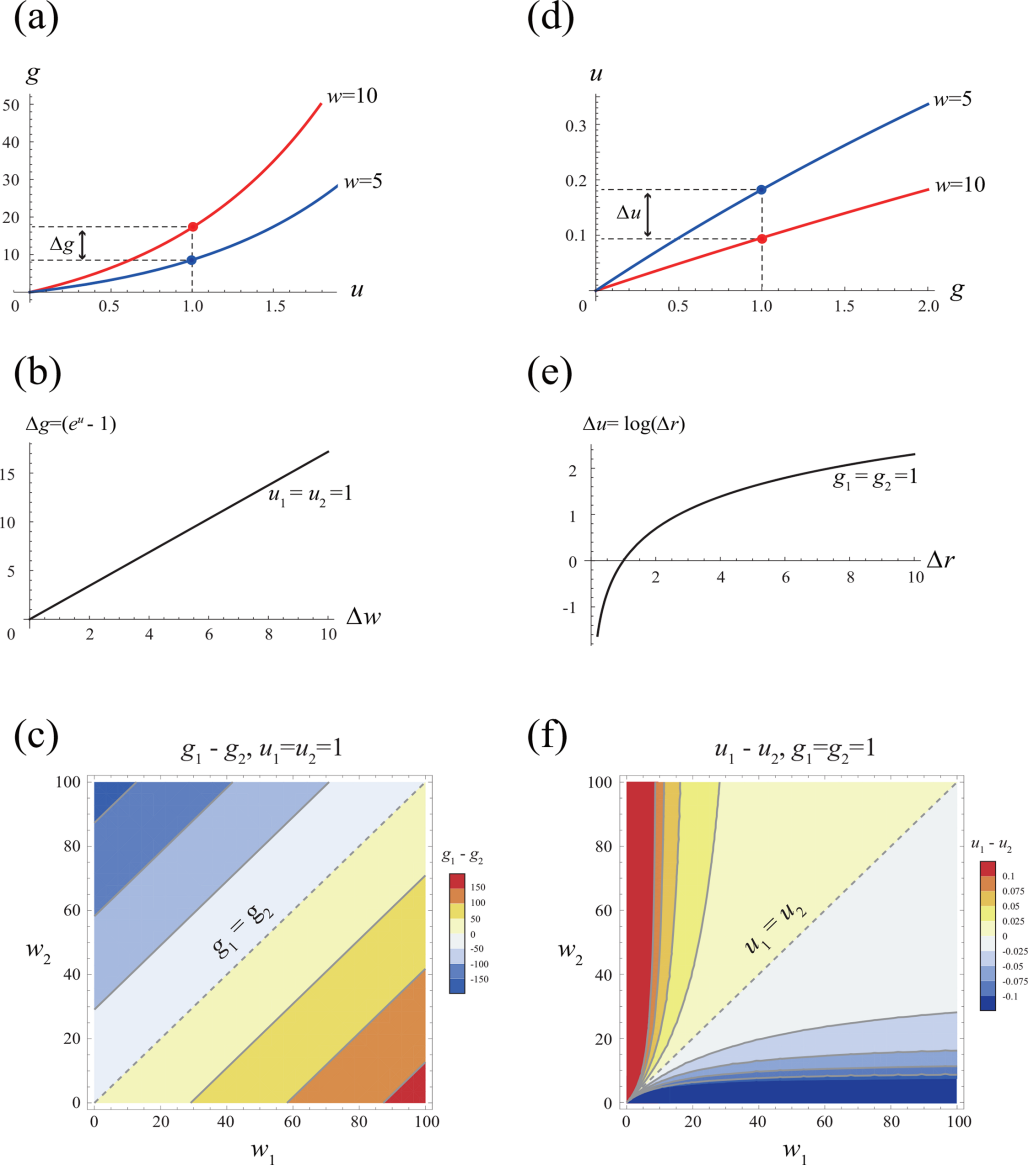
Comparison between the utility criterion and quantitative criterion. (a, b, c) The relationship between the gain and current wealth under the utility criterion (case 1, when *u*_1_ = *u*_2_ = 1). (d, e, f) The relationship between the utility and current wealth under the quantitative criterion (case 3). (a) The gain versus utility for both players, such that $g_{i}=w_{i}(e^{u_{i}}-1)$. *Δg* indicates the difference in *g* between the two players. (b) The difference in gain *Δg* (= |*g*_1_ –*g*_2_|) versus the difference in current wealth *Δw* (= |*w*_1_ –*w*_2_|). (c) Phase plane of *Δg* against *w*_1_ and *w*_2_. The dashed line indicate *Δg* = 0. (d) The utility versus both players’ gain, such that *u*_*t*_ = log{(*g*_*t*_ + *w*_*t*_)/*w*_*t*_}. *Δu* indicates the difference in *u* between the two players. (e) The difference in utility *Δu* (= |*u*_1_ –*u*_2_|) versus the difference in growth rate *Δr* (= *r*_1_/*r*_2_). (f) Phase plane of *Δu* versus *w*_1_ and *w*_2_. The dashed line indicates *Δu* = 0.

Now, we compare the quantitative differences between case 1 and case 3 (Table 1). The observed discrepancies in terms of both the utility (case 1) and quantitative (case 3) criteria increase with the difference in current wealth between the players (Fig 2). In the utility criterion (Fig 2A), the difference in the current gain *Δg* (= *g*_1_ –*g*_2_) depends linearly on the difference in current wealth *Δw* (= *w*_1_ –*w*_2_) (Fig 2B and 2C). In the quantitative criterion (Fig 2D), the difference in utility *Δu* (= *u*_1_ –*u*_2_) increases with the ratio of the multiplicative growth rates *Δr* (= *r*_1_/*r*_2_) of the two players, where *r* = (*g* + *w*) / *w* (Fig 2E and 2F). Note that if the current wealth of all players is equal, then we can preserve equality in both *g* and *u*, such that *g*_1_ = *g*_2_ and *u*_1_ = *u*_2_ simultaneously.

## Discussion

The current results demonstrate the drastic difference between the utility criterion and the quantitative criterion. Under the utility criterion, equal division means that the rich should obtain more than the poor. In contrast, under the quantitative criterion, both the rich and poor obtain the same amount of money (dollars), but their utility becomes different. Recent studies have shown that animals (e.g., human adults [32], human babies [33], and chimpanzees [34]) express a sense of fairness only when the reward is divided quantitatively in half. These studies suggest that these animals use the quantitative criterion in equal division.

This discrepancy in social interpretations could not have been resolved for more than a century because no utility can be derived unambiguously. From empirical studies of preferences in humans, utility (considered as perceptional quantity or psychophysical quantity) is known to correlate with the logarithm of the input (the stimulus quantity); this relationship is known as the Weber-Fechner Law [38–39]. Recent studies have also demonstrated that utility also depends on the current wealth of an individual decision maker [40], as in the analytically derived utility function applied in the current game. However, in traditional decision theory, the only method of estimating the utility function is to compare the preferences between two choices. Therefore, as Poincaré noted a century ago, we cannot even derive an approximate utility function mathematically [41]. The arbitrariness of utility thus cannot be avoided in any proposed utility functions. To avoid this problem, utility functions are treated as a black box without referring to the resources actually gained (rewards). Based on this problem, the resource dividends in traditional utility theory that are based on the utility criterion cannot be translated into actual amounts of resources.

The current results highlight a serious problem in repeated games. In a strict sense, traditional analyses of game theory are valid only when all players’ current wealth is equal. However, in any type of repeated games, players’ current wealth inevitably varies after each game. Thus, the utility function of a player varies in any sequential decisions, as long as his/her current wealth varies over time. Furthermore, the utility criterion is not applicable if the current wealth of players varies significantly. Therefore, traditional analyses are applicable only to the case of one-time decisions (games) in which the wealth of all players is equal. Note that the traditional equilibrium analyses become invalid even when the quantitative criterion is adopted. Thus, traditional analyses of game theory should be reevaluated in terms of DU. We should note that the utility criterion in game theory is still valuable because we have no alternative to estimate dynamic games. The traditional utility and game theory should be used as guidance for determining the exact (true) dynamic games. The definition of Nash equilibrium is still valid, and we expect that its analytical solution is also approximately true as long as the current wealth of players does not differ substantially.
